# Variation in Immune and Inflammatory Blood Markers in Advanced Melanoma Patients Treated with PD-1 Inhibitors: A Preliminary Exploratory Study

**DOI:** 10.3390/biomedicines13061378

**Published:** 2025-06-04

**Authors:** Lucica Madalina Bolovan, Marieta Elena Panait, Antonela Busca, Adina Elena Stanciu, Daniela Chiriac, Corina Elena Mihalcea, Camelia Mia Hotnog, Mihai Teodor Georgescu, Silviu Cristian Voinea, Virgiliu Mihail Prunoiu, Lorelei Irina Brasoveanu, Laurentia Nicoleta Gales

**Affiliations:** 1 Carcinogenesis and Molecular Biology Department, Institute of Oncology “Prof. Dr. Alexandru Trestioreanu”, 022328 Bucharest, Romania; madalina.bolovan@iob.ro (L.M.B.); adinaelenastanciu@yahoo.com (A.E.S.); daniela.chiriac@iob.ro (D.C.); corina_mihalcea81@yahoo.com (C.E.M.); 2 Cancer Biology Department, Institute of Oncology “Prof. Dr. Alexandru Trestioreanu”, 022328 Bucharest, Romania; me_panait@yahoo.com (M.E.P.); antonelabusca@gmail.com (A.B.); 3 Center of Immunology, “Stefan S. Nicolau” Institute of Virology, Romanian Academy, 030304 Bucharest, Romania; camelia.hotnog@virology.ro; 4 Biochemistry Department, University of Medicine and Pharmacy “Carol Davila” Bucharest, 050474 Bucharest, Romania; 5 Oncology Department, University of Medicine and Pharmacy “Carol Davila” Bucharest, 050474 Bucharest, Romania; mihai.georgescu@umfcd.ro (M.T.G.); laurentia.gales@umfcd.ro (L.N.G.); 6 Oncology Department, Institute of Oncology “Prof. Dr. Alexandru Trestioreanu”, 252 Fundeni Ave, 022328 Bucharest, Romania; 7 Oncological Surgery Department, University of Medicine and Pharmacy “Carol Davila” Bucharest, 050474 Bucharest, Romania; silviu.voinea@umfcd.ro; 8 Oncological Surgery Department, Institute of Oncology “Prof. Dr. Alexandru Trestioreanu”, 022328 Bucharest, Romania

**Keywords:** advanced melanoma, PD-1 inhibitors, peripheral blood immune cells, inflammatory markers, predictive melanoma biomarkers, cytokines

## Abstract

**Background:** Immune checkpoint inhibitors (ICIs) used for the treatment of advanced melanoma have yielded significant results, with long-term responses and improved survival rates, but not for all treated patients. Therefore, predictive biomarkers of response to ICI therapy have been intensively explored. Our study aimed to evaluate the dynamics of peripheral blood lymphocyte variation and their correlation with a set of related inflammatory factors in Nivolumab-treated advanced melanoma patients. **Methods:** The immunophenotypic assessment of peripheral blood immune cell subpopulations (CD3^+^, CD4^+^, and CD8^+^ T cells; CD19^+^ B cells; CD16^+^CD56^+^ NK cells; and CD4^+^/CD8^+^ ratio) was performed by the flow cytometry technique, concomitantly with a complete blood count; levels of S100, IL-6, and TNF-α proteins were quantified in serum by immunoassays, and lactate dehydrogenase (LDH) by a chemiluminescence assay. **Results:** Approximately 85% and 79% of patients recorded a trend of increasing levels of CD8^+^ lymphocytes and NK cells, respectively, during therapy. The percentage of NK cells negatively correlated with CD3^+^, CD4^+^, and CD19^+^ cells; the last three cell populations also established negative correlations with the inflammatory neutrophile/lymphocyte ratio (NLR). Furthermore, CD19^+^ cells were negatively correlated with the systemic inflammatory response index (SIRI) and systemic immune-inflammation index (SII). The evaluation of progression biomarkers showed that LDH levels directly correlated with IL-6 and S100 proteins, but no correlation was found with TNFα; IL-6 levels negatively correlated with percentages of CD3^+^, CD4^+^, and CD8^+^ lymphocytes. **Conclusions:** Variation in lymphocyte subpopulations during immunotherapy of advanced melanoma patients, associated with other cellular and/or molecular inflammatory markers, might provide insights about immune system response, but additional prospective studies are needed.

## 1. Introduction

Melanoma is the most aggressive form of skin cancer, characterized by genetic heterogeneity, unpredictable evolution, and resistance to systemic therapy. Currently, surgical excision remains the first-line therapeutic choice, added by radiotherapy, targeted therapy, and/or immunotherapy, depending on the stage of the disease, tumor location, and genetic profile [1]. In advanced melanoma, distant metastases remain the leading cause of melanoma-related deaths, with a 5-year survival rate of 23% [2,3,4]. However, new hope has emerged for metastatic melanoma patients since the clinical approval of monoclonal antibodies developed against immune checkpoints, such as cytotoxic T lymphocyte-associated antigen 4 (CTLA-4) in 2011 and, subsequently, programmed cell death protein-1 (PD-1) in 2014. The CTLA-4 immune checkpoint is a protein receptor expressed on activated or regulatory T cells, while the PD-1 receptor is expressed by activated T, B, and natural killer (NK) cells; both immune checkpoints provide inhibitory signals that downregulate the effector function to maintain the immune system homeostasis [5,6,7]. Anti-CTLA-4 (Ipilimumab) and anti-PD-1 (Pembrolizumab, Nivolumab) antibodies are commonly used agents in the immunotherapeutic practice, as immune checkpoint inhibitors (ICIs); their impact on the immune system significantly changed the prognosis of melanoma by increasing patients’ responsiveness, thus increasing the efficacy of therapy [8,9].

Therefore, the 5-year survival rate increased up to 52% in patients with advanced melanoma treated with the combination of Ipilimumab and Nivolumab [10,11]. However, despite the success of ICI therapy, not all tumors showed regression under immunotherapy, and equally durable clinical benefits were not shown for all patients: 20–40% of melanoma patients showed long-term benefits, and this proportion can reach up to 60% when ICIs are combined, but the rest of these patients developed resistance to therapeutic immune agents, mainly due to tumor heterogeneity and immune system cell variability or specific patient risks associated with a decrease in the overall survival rate [12,13,14,15,16]. Thus, to overcome tumor resistance, various studies have evaluated new potential markers that converge to discriminate between responders and non-responders to anti-PD-1/anti-CTLA-4 therapy, helping to stratify patients suitable for the most effective immunotherapy [17]. In this context, blood-based biomarkers seem to be reliable candidates due to their non-invasive nature. Immune cells, circulating tumor cells, DNA, RNA, and proteins represent valuable sources for the prediction of melanoma patients’ response to therapy [18,19].

The developed ICIs were designed to target the tumor–immune cell interface and were responsible for blocking the tumor cell signaling that inactivates immune system surveillance, thus allowing T cells to remain in an active state and attack the tumor cells [8,20,21]. However, the role of peripheral blood lymphocyte (PBL) subsets in controlling the immune system’s response to the aggressiveness of melanoma tumor cells is currently only partially understood, and the effects of ICIs on these cell subpopulations are being intensively studied [22,23,24].

The inflammatory process is one of the causes of the development and progression of different diseases such as diabetes, cardiovascular diseases, asthma, rheumatoid arthritis, allergies, chronic obstructive pulmonary disease, and various types of cancer, and many drugs directly target pro-inflammatory biomolecules in order to maintain the balance between pro- and anti-inflammatory effects. Also, novel innovative inflammation-related biomarkers are investigated to reflect the state of systemic inflammation [25,26,27]. Recent clinical studies associate several white-blood-cell-based inflammatory indices with the intensity of inflammatory reactions, disease severity, or therapy efficacy: (a) the systemic immune-inflammation index (SII), a prognostic factor; (b) the systemic inflammatory response index (SIRI), a predictive biomarker of therapeutic response; and (c) the neutrophil-to-lymphocyte ratio (NLR), monocyte-to-lymphocyte ratio (MLR), and platelet-to-lymphocyte ratio (PLR), known as markers of immune system homeostasis and prognostic indicators [28,29,30,31,32]. The assessment of lymphocyte, neutrophil, monocyte, and platelet counts has the advantage of being easily accessible, non-invasive, and cost-effective and quickly provides data that could suggest the inflammatory status, which is useful in clinical decisions for managing melanoma patient therapy.

On the other hand, the inflammatory processes that are sustained by the inflammatory-related cells, cytokines, and chemokines within the tumor microenvironment (TME) play a major role in tumor cell progression or response to therapy. Immunological signaling proteins, such as the cytokines interleukin-6 (IL-6) and tumor necrosis factor (TNF-α), are among the various inflammatory factors involved in the therapeutic resistance due to their pro-inflammatory effects, acting either at the local cellular level or remotely in soluble forms [33,34]. The persistent inflammatory state may support and stimulate tumorigenesis, such that overexpression of inflammatory mediators induces an inflammatory cascade leading to an impaired immune response that favors the promotion of invasion and metastasis [35,36]. Therefore, one of our objectives was to investigate the potential relationship established between IL-6 and/or TNF-α and the white-blood-cell-based inflammatory indices, which could provide valuable information about the efficacy of melanoma treatment.

In addition to the immune system parameters described above (immune indices and cytokine release), knowledge of serum levels of melanoma tumor markers, such as serum lactate dehydrogenase (LDH) and S100, could provide useful information regarding the metabolic activity of tumor cells and tumor progression (tumor growth and tumor burden) by their individual or corroborated assessment during immunotherapy [37,38,39,40,41,42,43]. LDH is a well-known metastatic melanoma biomarker, and since 2009, it has been included as a recommendation in melanoma staging guidelines; recent reports have shown that elevated baseline values of LDH are correlated with poor survival and response rates in melanoma patients treated with anti-PD-1. S100 proteins are also routinely assessed both at the serum level, with a higher melanoma specificity than LDH, and at the tissue level, as a specific and reliable immunohistochemical marker in malignant melanoma [44].

Moreover, previous studies conducted by our group on melanoma patients who underwent surgery at the “Prof. Dr. Alex. Trestioreanu” Institute of Oncology in Bucharest, Romania, showed significant correlations between the levels of S100, gp100 (HMB45), or MelanA tissue expression and the soluble markers S100B and MIA [37]. Following that study, we selected a group of patients diagnosed with advanced melanoma, eligible for immunotherapeutic approaches, to perform further studies focused on their immunological status during the treatment dynamics.

Therefore, the current study aims to (i) measure and monitor the variation in lymphocyte cell subpopulations, such as CD3^+^ T lymphocytes, CD19^+^ B lymphocytes, CD3^+^CD4^+^ T helper/inducer lymphocytes, CD3^+^CD8^+^ T suppressor/cytotoxic lymphocytes, helper/suppressor T-lymphocyte ratio (CD4^+^/CD8^+^), and CD16^+^CD56^+^ natural killer (NK) cells, during Nivolumab therapy in the selected melanoma patients and (ii) measure and monitor the variation in the inflammatory indices of white blood cells (iii) to investigate the potential correlations between the expression of lymphocyte cell subsets, the inflammatory indices of white blood cells, and specific soluble markers associated with melanoma tumor progression, such as LDH, S100B, IL-6, and TNF-α.

## 2. Materials and Methods

### 2.1. Patients and Study Protocol

The exploratory study group who underwent immunotherapy involved 20 patients (13 F/7 M, mean age: 57.5 ± 12.6 years) selected from a larger cohort of 176 melanoma patients (78 F/98 M, mean age: 56.2 ± 12.1 years) who underwent surgery and were retrospectively evaluated in a previous study regarding the possible correlations between the soluble forms of melanoma-specific markers, S100B and MIA proteins, and the tissular expression of S100, gp100 (HMB45), and MelanA (MART-1), currently used in the differential melanoma diagnosis. The patients included in the previous study were enrolled between 2016 and 2022 and diagnosed with cutaneous melanoma at different stages of the disease, as follows: stage I (*n* = 27), stage II (*n* = 53), stage III (*n* = 74), and stage IV (*n* = 22), confirmed by their histopathological analysis. The control group was represented by 56 healthy blood donors (25 M/31 F) [37].

The selected melanoma patients were followed throughout the course of the immunotherapy after being diagnosed with advanced cutaneous melanoma (CM), stage III (*n* = 8) and stage IV (*n* = 12), classified according to the 8th Edition American Joint Committee of Cancer (AJCC) melanoma staging system, registered and treated with anti-PD-1 antibodies, between 2020 and 2022 at the “Prof. Dr. Alex. Trestioreanu” Institute of Oncology in Bucharest, Romania. The eligible patients cumulatively met the following inclusion criteria: (i) patients in stages III and IV of the disease, with metastases or unresectable tumors; (ii) patients under PD-1 inhibitor therapy, with Nivolumab; (iii) age over 18 years; (iv) availability of patient’s medical and treatment history. The exclusion criteria were represented by any history of previous immune, inflammatory, or cardiovascular diseases or diabetes.

The immunotherapy consisted of the administration of Nivolumab, 240 mg every 2 weeks or 480 mg every 4 weeks. The patients were evaluated by imaging according to the criteria of the Response Evaluation Criteria in Solid Tumors version 1.1 (RECIST 1.1) and adapted to the protocol of Nivolumab administration. The treatment was well tolerated in our cohort, with minor and moderate toxicities, mainly endocrine. It was stopped exclusively for disease progression, and no differences were found between the regimens treated every 2 or 4 weeks. Clinical information on the patients’ condition was collected throughout this study, which included the age, sex, tumor location and histological characteristics, disease stages, ICI therapy cycles, and imaging investigations. The control group consisted of 20 healthy volunteers, (12 F/8 M, mean age: 47.8 ± 8.5 years) without any history of malignancy or immune or inflammatory diseases.

### 2.2. Blood Sampling and Methods

Peripheral blood samples from patients with advanced melanoma were collected throughout this study in the treatment dynamics, before each Nivolumab cycle administration. The number of samples taken from each patient ranged from 3 to 13 samples, depending on the evolution of the disease, and the samples were collected one day before each Nivolumab cycle, concomitant with complete blood count (CBC) and biochemistry samples. The CBC was performed on the entire cohort of samples to evaluate the peripheral blood cell populations, followed by immunophenotyping using the flow cytometry technique to assess lymphocyte subpopulations. We also investigated the levels of two soluble melanoma markers (LDH, S100B) and two inflammatory cytokines (IL-6, TNF-α) in 60 samples selected from the study database, at 3-month intervals, depending on the therapy time points (3 samples per patient).

The peripheral blood was drawn in sterile 3 mL BD Vacutainer K_2_EDTA anticoagulant tubes from the cubital vein. The probes were used within a maximum of 6 h from peripheral blood collection for both the analysis of blood count parameters (neutrophils (Neus), monocytes (Monos), lymphocytes (LYMs), and platelets (PLTs)) and immunophenotyping assays [25]. In addition, 5 mL blood samples were collected in tubes containing serum clot activators and further used to detect the serum levels of soluble LDH, S100, IL-6, and TNF-α proteins. The sera, obtained by centrifugation (15 min/1000× *g* at 4 °C), were aliquoted and stored at −80 °C until use. The blood samples from the control group were also stored and processed in the same manner as the samples from melanoma patients [37].

### 2.3. Complete Blood Counting (CBC)

The CBC analysis was performed with ADVIA 2120i Hematology System (Siemens Healthineers AG, Munich, Germany), which provided data results expressed both as absolute and percentage values for lymphocytes, neutrophils, monocytes, and platelets, and further used to assess the white-blood-cell-based inflammatory indices/ratios. The levels of inflammatory indices NLR, MLR, and PLR were obtained by dividing the absolute neutrophil, monocyte, and platelet counts by the absolute lymphocyte count. The systemic immune-inflammation index (SII) and the systemic inflammatory response index (SIRI) integrated four types of immune cells (Neus, Monos, Lyms, PLTs), and were calculated using the following formulas [25]:

SII = PLT count [×10^9^/L] × Neu count [×10^9^/L]/LYM count [×10^9^/L]

SIRI = Neu count [×10^9^/L] × Mono count [×10^9^/L]/LYM count [×10^9^/L].

The reference intervals (RIs) for inflammatory markers were (a) NLR: 0.78–3.53, (b) MLR: 0.122–0.474 for females and 0.136–0.505 for males, (c) PLR: 61–239 [45,46], (d) SII: 189–1168, and (e) SIRI below 1.26 [47,48,49,50].

### 2.4. Immunophenotyping

The lymphocyte-subset-specific receptors were identified by two-color direct immunophenotyping and assessed by acquisition and further analyses using the flow cytometry approach and specific pieces of software. The procedure involved the use of erythrocyte-lysed whole blood for each detection tube to assess the immune cells; the cell subpopulations were characterized using the BD Simultest^TM^ IMK Plus Kit (BD Biosciences, San Jose, CA, USA) [51].

The kit contains several pairs of murine anti-human monoclonal antibodies directed against cell-surface markers and conjugated with FITC and PE fluorochromes (BD Biosciences, San Jose, CA, USA). Briefly, 100 μL/tube of a fresh peripheral blood sample was mixed with 20 μL of specific fluorescent monoclonal antibodies, which included the following: (1) BD Leucogate (CD45/CD14): FITC-labeled anti-CD45, clone 2D1, for identification of leucocytes and PE-labeled anti-CD14, clone MϕP9, for identification of monocytes; (2) Control: FITC-labeled IgG_1_, clone X40, and PE-labeled IgG_2_a, clone X39; (3) CD3/CD19: FITC-labeled anti-CD3, clone SK7, for identification of T lymphocytes and PE-labeled anti-CD19, clone 4G7, for identification of B lymphocytes; (4) CD4/CD8: FITC-labeled anti-CD4, clone SK3, for identification of CD4 lymphocytes and PE-labeled anti-CD8, clone SK1, for identification of CD8 lymphocytes; (5) CD3/CD16+CD56: FITC-labeled anti-CD3, clone SK7, for identification of T lymphocytes and PE-labeled anti-CD16, clone B73.1/PE-labeled anti-CD56, clone MY31, for identification of NK cells.

Then, stained samples were treated with BD FACS lysing solution to lyse erythrocytes and washed twice with PBS (300× *g* for 5 min/RT). The data were acquired with CellQuest software version 7.5.3., BD Biosciences, San Jose, CA, USA) installed on a FACSCalibur flow cytometer (Becton Dickinson Immunocytometry Systems, San Jose, CA, USA) and analyzed with CellQuest and WinMDI 2.9 software. The results are presented as percentages of positive cells in the lymphocyte acquisition gate (10^4^ events) set by using the BD Leucogate (CD45/CD14) tube. In addition to the percentage assessment, the absolute cell count was calculated using the white blood cell (WBC) count and Ly percentage from a differential white count, provided by standard laboratory procedures (CBC) [49].

Absolute count of a lymphocyte subset = Ly subset (%)/100 × Ly (%)/100 × WBC/µL

### 2.5. Detection of Soluble Markers

Immunoassays were used to detect several soluble markers: soluble S100B protein was quantified using the CanAg S100 EIA kit (Fujirebio Diagnostics AB, Göteborg, Sweden), which allows the specific binding to the S100A1B and S100BB epitopes expressed on S100B protein; serum levels of the cytokines IL-6 and TNF-α were assessed using Human IL-6 or Human TNF-alpha Uncoated ELISA kits (Invitrogen, Bender MedSystems GmbH, Vienna, Austria) by attaching the proteins of interest to biotin-conjugated specific antibodies coupled to the streptavidin–HRP enzyme complex. The standard curves were calculated linearly according to the kit recommendations. The upper limit of normal concentration for serum S100B was 90 ng/L, as established by the kit manufacturer. The cut-off value used for IL-6 was ≤5.186 pg/mL [52], while for TNF-α, it was ≤8.1 pg/mL [53]. The LDH values were detected by a chemiluminescence assay with the ALINITY ci-series System (Abbot, IL, USA) with a reference range of 125–220 U/L [54,55,56].

### 2.6. Statistical Analysis

The experimental data were analyzed using several software packages, including Microsoft Office Excel 2007 and GraphPad Prism 9.3.0 software (GraphPad Software Inc., La Jolla, CA, USA), and presented as mean and median values, standard deviation (SD), quartile range (QR, 25th–75th%), and/or percentages (%). The Pearson correlation coefficient (*r)* was used to determine the associations between the parameters under study. The *r* values showed a strong correlation for ±0.5 ≤ *r* ≤ ±1, a moderate correlation for ±0.3 ≤ *r* ≤ ±0.49, and a low correlation for *r* ≤ ±0.29. The statistical significance was presented as *p*-values, with a threshold of less than 0.05, and * *p* < 0.05, ** *p* < 0.01, and *** *p* < 0.001 were considered statistically significant.

## 3. Results

### 3.1. Evaluation of Lymphocyte Subpopulations in Patients with Advanced Melanoma

Our main objective was to assess the variation in the expression of lymphocyte subpopulations during immunotherapy and their relationship with other biomarkers of tumor progression in 104 samples from 20 melanoma patients, selected as described in Section 2, out of 176 patients previously enrolled and followed at the “Prof. Dr. Alex. Trestioreanu” Institute of Oncology in Bucharest [37].

The selected patients were classified in stages III and IV and eligible for anti-PD-1 therapy. The characteristics of the advanced melanoma patients included in our study are described in Table 1.

The CD3^+^, CD4^+^, CD8^+^, CD19^+^, and CD16^+^CD56^+^ immune cell markers were evaluated by an immunophenotyping assay in the peripheral blood, using data acquisition and analyses by the flow cytometry technique. We compared the values of immunophenotyping markers associated with the peripheral blood lymphocytes, measured in both Nivolumab-treated melanoma patients and healthy control volunteers. Statistically significant differences were obtained between the two groups in terms of cell subpopulation percentages for CD3^+^*,* CD4^+^, CD19^+^, and CD16^+^CD56^+^ cells (*p* < 0.05), but no statistical changes were recorded for the CD8^+^ subset and CD4^+^/CD8^+^ ratio (*p* > 0.05). Analyzing the median values and the quartile range of these samples, we observed that the median values of CD3^+^ cells were similar in the melanoma and control groups, while the quartile range was higher in the melanoma group. The CD4^+^ subset showed a decrease in the median values associated with an increase in the range limits; the CD8^+^ subset and CD4^+^/CD8^+^ ratio slightly decreased compared to the normal values; in contrast, CD19^+^ and CD16^+^CD56^+^ cells showed higher median and range values than the control group (Table 2). Our findings suggested that the percentages of NK and CD19^+^ cells increased and the percentages of CD3^+^ and CD4^+^ cells decreased in melanoma patients compared to the control group. The Supplementary Materials (Table S1) shows the percentage values of the studied markers in the blood samples from three melanoma patients, compared to a normal control, determined by immunophenotypic analysis by flow cytometry, using WinMDI 2.9 software. The data are presented both as dot plots for double-stained markers and as overlaid histograms for each marker (Supplementary Materials, Table S1).

From these data, it seems that the group of melanoma patients showed slight increases in the percentages of B lymphocytes and NK cells during anti-PD-1 treatment, which were statistically different compared to the results in the healthy group. The T lymphocyte subsets expressed variations from normal values based on the quartile range, explaining the statistical differences in CD3^+^ and CD4^+^ T lymphocytes compared to controls.

### 3.2. Dynamic Variation in Peripheral T Lymphocyte Subsets, B Lymphocytes, and NK Cells During Immunotherapy

**Figure 1:**
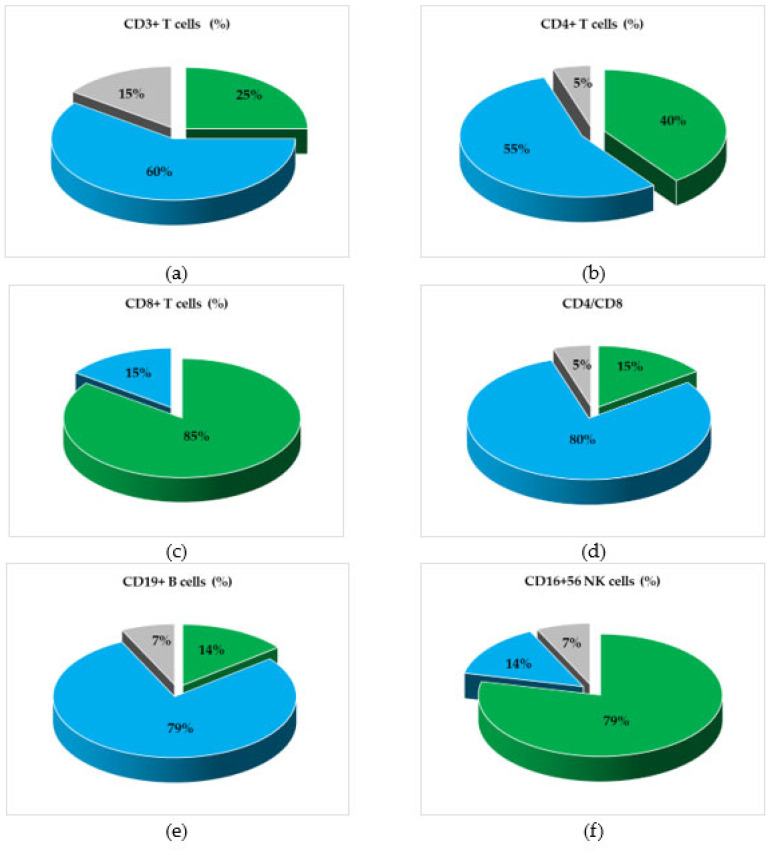
Graphical representation of patients’ categories, divided according to the variation trends (increasing, decreasing, or stationary) of the levels of cellular subpopulations, during treatment with Nivolumab. Percentages of Nivolumab-treated patients’ categories, divided according to the variation trends of (**a**) total T lymphocytes (CD3^+^), (**b**) helper/inducer T lymphocytes (CD3^+^CD4^+^), (**c**) suppressor/cytotoxic T lymphocytes (CD3^+^CD8^+^), (**d**) the helper/suppressor T cell ratio (CD4^+^/CD8^+^), (**e**) total B lymphocytes (CD19^+^), and (**f**) natural killer (NK) cells (CD3^+^/CD16^+^CD56^+^). Color legend: percentages of patients with stationary (grey), increasing (green), or decreasing (blue) status of cellular subsets.

Immune cells were analyzed in the dynamics of Nivolumab treatment, and their variation trend was determined starting with the first collected sample for each melanoma patient throughout this study. Figure 1 presents the percentages of patients, divided into three trends of variation (increase, decrease, or stationary) for each lymphocyte marker under study, and shows a trend of decreasing values for CD3^+^ T cells in 60% of the patients and for the CD4^+^/CD8^+^ ratio in 80% of the cases. However, the above markers were expressed in melanoma patients below the minimum normal limits in 30% and 35% of the patients, respectively. In addition, the percentage of CD19^+^ B cells decreased in 79% of the patients but remained within normal limits; in contrast, the CD16^+^CD56^+^ NK cells increased in 79% of the cases, with 15% of patients having values higher than normal. The downward trend in the CD4^+^/CD8^+^ ratio was a consequence of the increase in the percentage of CD8^+^ T cells and the decrease in CD4^+^ T cells; the decrease in CD19^+^ B lymphocytes was accompanied by an increase in the NK cell percentage, with the Pearson *r* correlation coefficient showing a significant negative correlation (*r* = −0.498, *p* < 0.001), as mentioned in the Supplementary Materials (Table S2). The clinical evidence that differentiates the responder group from the non-responder patients is not presented and analyzed in the current study, but we noticed that all patients who registered therapeutic benefits throughout this study, evaluated according to RECIST 1.1 criteria, were characterized by increased trends for NK cells and CD8^+^ T cells, while the trends for CD19^+^ B cells varied within normal ranges.

### 3.3. Correlation Between Levels of Immune Cells and Systemic Inflammatory Markers

Correlation analyses were further performed to investigate the potential correlations established in blood samples of melanoma patients during Nivolumab therapy between the levels of immune cell subpopulations (CD3^+^, CD4^+^, CD8^+^, CD19^+^, CD16^+^CD56^+^), assessed by flow cytometry, and the systemic inflammatory markers (SII, SIRI, NLR, MLR, PLR). In addition, Table 3 presents the hematological data obtained after CBC assessment of peripheral blood populations for the samples selected at T1, T2, and T3, at intervals of 3 months for all patients, based on which the inflammatory ratios/indices were calculated. From these data, it follows that NLR, PLR, and SII showed differences between samples collected at T1 and T3, and the median values after six months of therapy were lower compared to the first measurement, while the median values of MLR and SIRI increased slightly due to the median value of monocytes, which was higher at T3 than at T1. The results suggest that during this period, the inflammatory state was influenced by the treatment, but a reliable conclusion can only be drawn by evaluating each patient and supported by additional clinical evidence.

Moreover, in the entire sample cohort (*n* = 104), we observed that CD3^+^ T cells, CD4^+^ T cells, and CD19^+^ B cells have a moderate negative correlation with NLR. Furthermore, we found that CD19^+^ B cells are moderately negatively correlated with the SIRI (*r* = −0.316, *p* < 0.05) and SII (*r* = −0.315, *p* < 0.05) indices. A strong positive correlation was obtained between SIRI and SII (*r* = 0.868, *p* < 0.001); in addition, each of these two parameters was strongly correlated with the NLR, MLR, and PLR ratios, as shown in Figure S1 and Table S2 (Supplementary Materials). The analysis of the percentages of lymphocyte subpopulations revealed that the percentage of CD3^+^ T lymphocytes was strongly positively correlated with the percentages of the CD4^+^ (*r* = 0.475, *p* < 0.001) and CD8^+^ (*r* = 0.585, *p* < 0.001) subpopulations, while a strong negative correlation (*r* = −0.820, *p* < 0.001) was found between the percentages of CD3^+^ T cells and CD16^+^CD56^+^ NK cells.

The levels of CD19^+^ B lymphocytes were moderately positively correlated with the CD4^+^/CD8^+^ T cell ratio (*r* = 0.329, *p* < 0.01) and strongly negatively correlated with both the levels of CD8^+^ T cells (*r* = −0.505, *p* < 0.001) and CD16^+^CD56^+^ NK cells (*r* = −0.498, *p* < 0.001). As expected, the CD4^+^/CD8^+^ ratio was strongly positively correlated with the CD4^+^ subpopulation (*r* = 0.656, *p* < 0.001) and negatively correlated with the CD8^+^ subpopulation (*r* = −0.777, *p* < 0.001). The percentage of CD4^+^ T cells was moderately negatively correlated with the level of CD16^+^CD56^+^ NK cells (*r* = −0.443, *p* < 0.001). As a conclusion, it could be noticed that the percentage of NK cells was negatively correlated with the percentages of CD3^+^ T cells, CD4^+^ T cells, and CD19^+^ B cells. The comprehensive images of the statistical analyses between the immune parameters under study, represented by Pearson *r* correlation coefficients and *p*-values, are presented in Figure S1 and Table S2 (Supplementary Materials).

### 3.4. Assessment of Cytokine Release in Nivolumab-Treated Melanoma Patients

**Figure 2:**
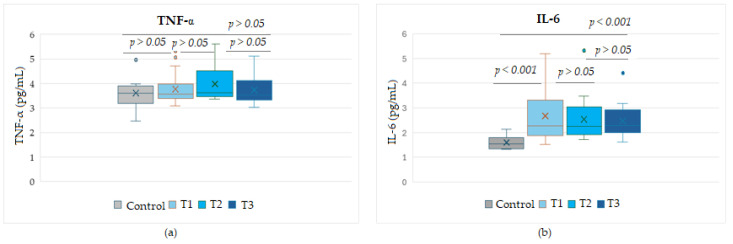
Circulating levels of the cytokines TNF-α (**a**) and IL-6 (**b**) during the course of Nivolumab therapy, for the time points T_1_, T_2_, T_3_. T1–T3, sampling times from Nivolumab-treated melanoma patients, taken at 3-month intervals.

Due to their major role in the tumor cell progression, the levels of the pro-inflammatory cytokines TNF-α and IL-6 were evaluated in dynamics during Nivolumab treatment in three selected serum samples (T1–T3, timepoints of Nivolumab treatment) from each patient at 3-month intervals. Analyzing the variation in the two proteins, we found no significant difference between the levels of TNF-α in Nivolumab-treated patients compared to the control group. In contrast, the levels of soluble IL-6 detected in samples collected at any timepoint from melanoma patients were significantly higher than controls (*p* < 0.01). However, a decrease in IL-6 levels was observed with an increase in the number of treatment cycles, from T1 to T3, even though no statistical significance (*p* > 0.05) was found between these time points, suggesting a heterogeneity between patients at T1 that decreases with the increasing number of Nivolumab cycles in melanoma patients (Figure 2).

**Figure 3:**
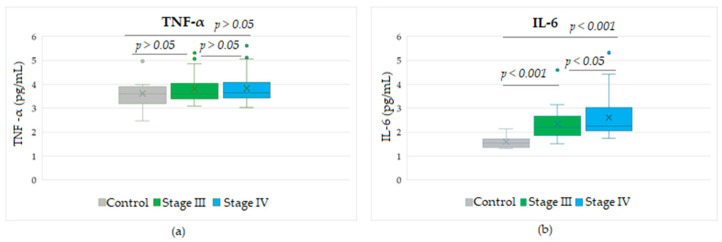
Circulating levels of the cytokines TNF-α (**a**) and IL-6 (**b**) in patients with advanced stage III or IV melanoma during treatment with Nivolumab. Levels of TNF-α or IL-6 were detected in the entire cohort of peripheral blood samples, taken at 3 time points at 3-month intervals, from the start of therapy.

Figure 3 shows the expression levels of soluble cytokines detected in stages III and IV melanoma patients and compared with control group values. While the TNF-α protein did not show significant differences between any melanoma stage compared to the control group or between stages (*p* > 0.05), soluble IL-6 levels were statistically different either between melanoma patients and the control group (*p* < 0.001) or between the two advanced stages (*p* < 0.05). It was observed that IL-6 values in stage IV patients were higher than values in stage III patients, even though the median values were comparable, but the SD variation was increased in stage IV patients.

### 3.5. Correlation Analyses Between Systemic Inflammatory Markers and Soluble Tumor Markers

Due to the current increased interest in the significance of the systemic inflammatory indices SII and SIRI as prognostic and predictive factors in many malignant diseases, our study focused on their simultaneous evaluation with a set of circulating biomarkers involved in different signaling pathways that have a major role in melanoma progression, such as the pro-inflammatory cytokines IL-6 and TNF-α, S100, and LDH. Levels of soluble biomarkers were detected in samples collected from melanoma patients treated with Nivolumab, as described in the Section 2, and the obtained values were further subjected to statistical analyses. The statistical results and correlation parameters for each analyzed biomarker in samples collected at 3-month intervals are presented in Table 4 and Figure 2

**Figure 4:**
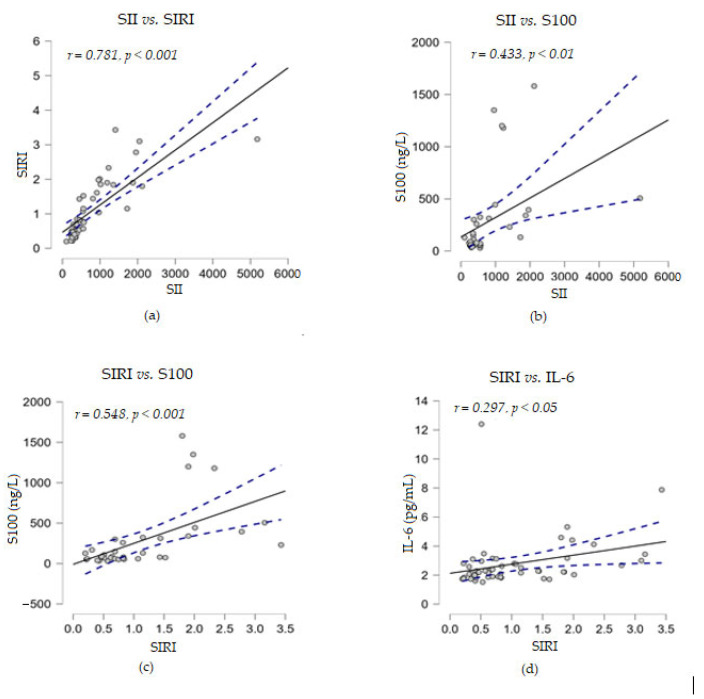
Correlation analyses between systemic inflammatory markers and several soluble melanoma markers: (**a**) SII vs. SIRI; (**b**) SII vs. S100; (**c**) SIRI vs. S100; (**d**) SIRI vs. IL-6; (— fitted linear regression curve; --- equality line). SII, systemic immune-inflammation index; SIRI, systemic inflammatory response index; IL-6, interleukin-6.

The statistical analyses between the inflammation indices SII and SIRI showed a significant direct strong correlation (*r* = 0.868, *p* < 0.001). When SII and SIRI were analyzed for their potential correlation with levels of the four soluble biomarkers detected in melanoma patients, we observed a moderate positive correlation between SII and S100 proteins (*r* = 0.433, *p* < 0.01), while SIRI was strongly positively correlated with this marker (*r* = 0.548, *p* < 0.001). A low significant direct correlation was found between SIRI and the levels of soluble IL-6 (*r* = 0.297, *p* < 0.05), but not for SII (Figure 4).

**Figure 5:**
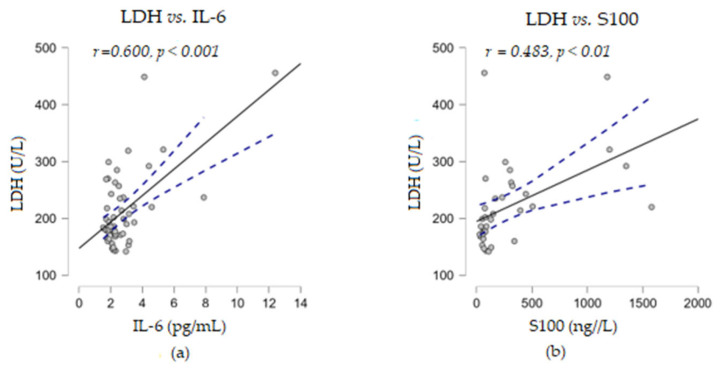
Correlation analyses between LDH and other soluble melanoma markers: (**a**) LDH vs. IL-6; (**b**) LDH vs. S100 (— fitted linear regression curve; --- equality line). LDH, lactate dehydrogenase; IL-6, interleukin 6.

Furthermore, no correlation was found between the above two immune indices with the levels of soluble TNF-α or LDH proteins. When correlation analyses were performed between the levels of the soluble markers under study, we observed that LDH established a strong positive correlation with the cytokine IL-6 (*r* = 0.600, *p* < 0.001) and a moderate positive correlation with S100 (*r* = 0.483, *p* < 0.01) (Figure 5). Considering these positive associations of the three parameters, the corroboration of their high levels could suggest a risk of resistance to immunotherapy.

**Figure 6:**
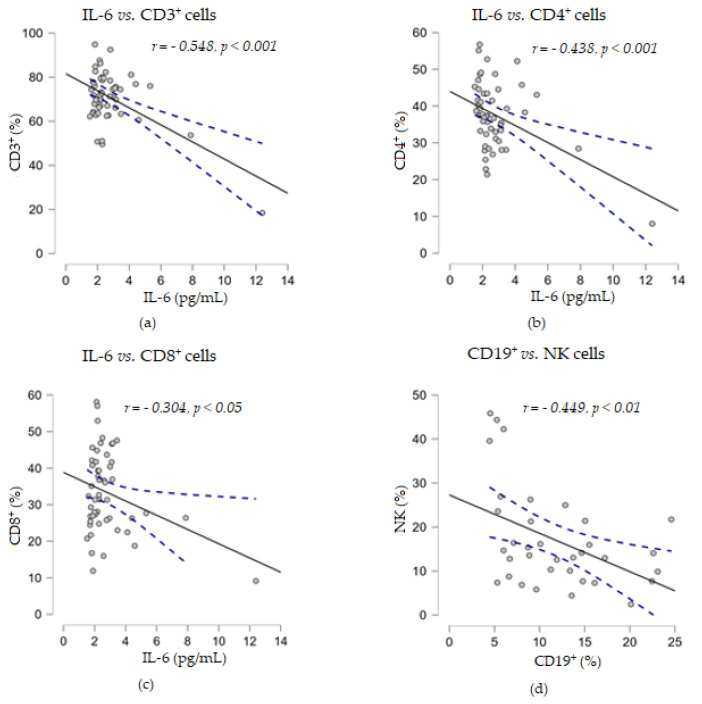
Correlation analyses between soluble cytokine IL-6 levels and the percentages of T lymphocyte subsets ((**a**) IL-6 vs. CD3^+^ cells; (**b**) IL-6 vs. CD4^+^ cells; (**c**) IL-6 vs. CD8^+^ cells) or between the percentages of CD19^+^ and NK cells (**d**) (— fitted linear regression curve; --- equality line). IL-6, interleukin 6.

In addition, the experimental results showed that IL-6 levels were strongly negatively correlated with the percentage of CD3^+^ T cells (*r* = −0.548, *p* < 0.001) and moderately negatively correlated with CD4^+^ (*r* = −0.438, *p* < 0.001) and CD8^+^ (*r* = −0.304, *p* < 0.01) T cells, while no statistical relationship was found between soluble TNF-α levels and any of the analyzed percentages of cell subpopulations (Figure 6).

## 4. Discussion

Significant achievements have been continuously made to improve treatments for cancer, but this disease has continued to place a great burden on healthcare systems worldwide. In addition to classical treatments like surgery, polychemotherapy, and radiotherapy, new medical agents such as angiogenesis inhibitors or checkpoint inhibitors have been introduced as treatment options in oncology clinics [5,55,56]. Currently, during the clinical management of melanoma, several biomarkers have been explored in addition to the histological characteristics (tumor thickness, ulceration, mitotic rate, anatomic site, sentinel lymph node, metastasis). Among them, the following are of great importance: nucleic acids (circulating tumor DNA, RNA, microRNA) or various genomic alterations (BRAF, NRAS, NF1, c-Kit); circulating cells (tumor cells, peripheral blood CD19^+^B lymphocytes, CD4^+^CD69^+^ T cells^+^, NK cells, monocytic myeloid-derived suppressor cells—(Mo)–MDSCs), cell indices (NLR, SII), and molecules/metabolites (LDH, S100B, MIA, cytokines, TIMP1, TGF-β, PD-L1). However, some of these biomolecules have specificity for melanoma tissue but are not limited to this disease [37,57,58,59,60,61,62].

The use of ICI therapy for advanced melanoma has provided a bridge that achieves significant survival gains, from a median of 6–9 months before the inclusion of immunotherapy in the treatment protocol to nearly 6 years for patients with advanced melanoma stages as a result of a combination of ICI agents [63,64]. However, due to the tumor heterogeneity and immunosuppressive environment of melanoma cells, the resistance of all the patients who progressed during immunotherapy remains a significant challenge to overcome [14,18,65].

The tumor microenvironment is highly immunosuppressive, establishes a strong interdependent relationship with tumor cells, and becomes fundamental for tumor initiation, cell growth, and invasion processes. In this regard, the relation between the expression of immune cells and/or their products (cytokines) as soluble signaling proteins that regulate the immune response or cell-to-cell communication in synergistic or antagonistic ways with other circulating biomarkers involved in the tumor cell progression through different signaling pathways could provide a complex view of these molecular factors competing as mediators in the tumor invasion process [66,67]

Previous studies conducted at the Institute of Oncology in Bucharest focused on measuring the efficacy of cancer treatments, considering the potential influence of different peripheral blood lymphocyte subpopulations on tumor progression, immune-related inflammatory indices, in relation to clinical and pathological parameters, and their response to classical treatments [25,50,68].

Considering the cellular communications and the need to improve the area of specific predictive and/or prognostic markers for therapies involving PD-1 inhibitors, in this study, we analyzed the variation in a set of surface markers of circulating immune cells, such as CD3^+^, CD4^+^, and CD8^+^ T cells; CD19^+^ B cells; CD16^+^CD56^+^ NK cells; and the CD4^+^/CD8^+^ T cell ratio, and their potential correlation with the inflammatory indices SII, SIRI, NLR, MLR, and PLR. In addition, we investigated several tumor progression proteins that are frequently assessed in the clinical evaluation of melanoma, such as LDH, S100, IL-6, and TNF-α.

The evaluation of the variation in immune cell dynamics during the PD-1/PD-L1 complex blockade showed that cytotoxic CD8^+^ T cells and NK cells registered moderately increased levels, and percentage values higher than normal limits were found in 30% of cases for CD8^+^T cells and 15% of cases for NK cells. We noticed that no correlation was found between the levels of these cell subpopulations, and the percentage of NK cells established a negative correlation with CD3^+^, CD4^+^, and CD19^+^ cells. NK cells have a major role in initiating or enhancing the immune response together with CD8^+^ and CD4^+^ T cells, either directly, due to their cytotoxic effects, or indirectly by regulating the composition and activity of infiltrating immune cells, the direction in which cell expression could suggest the state of the immune cells during treatment [19,69,70,71,72]. Moreover, based on this crosstalk between NK and T cells, we found a negative correlation between circulating NK cells and CD19^+^ B cells throughout this study. Even though the levels of CD19^+^ B cells tended to decrease, the values were found to be within normal limits for all the patients, supporting immune stability [73,74,75].

Recently, many studies have intensively debated the pro- and anti-tumor potential of B cells, suggesting that differentiated B lymphocytes are positively correlated with ICI response or that certain B-cell subsets are often correlated with immune-related adverse events [76]. On the other hand, according to other study., circulating B lymphocyte subsets, besides other cells like T cells, macrophages, dendritic cells, and mast cells, can produce inflammatory cytokines, such as IL-6 and TNF-α, which induce the checkpoint blockade failure, thus contributing to tumor growth and being associated with the lack of response and poor outcome of melanoma patients treated with anti-CTLA-4. These B cells appear to be different from tertiary-lymphoid-structure-associated B cells, but further studies are needed to identify the immunophenotype of these B-cell subsets and characterize their cellular functions [77].

In consideration of the production of these cytokines and their involvement in melanoma resistance to immunotherapy, we evaluated the blood levels of the cytokines TNF-α and IL-6. The results showed a negative correlation of IL-6 levels with the percentages of CD3^+^, CD4^+^, and CD8^+^ T cells. Furthermore, IL-6 levels decreased with the increase in the number of treatment cycles, suggesting that the production of these pro-inflammatory cytokines was influenced by the immune system response under Nivolumab therapy. However, further studies and a larger cohort of patients are needed to add statistical power to our preliminary data. Previous scientific works, such as the article of Hirano et al. [78], described the molecular mechanism by which the cytokine IL-6 inhibits T-cell immunity against tumors and the complexity of IL-6 cellular signaling, highlighting how the immune system regulates the homeostasis of CD4^+^ or CD8^+^ cell subpopulations in a manner dependent on the IL-6-STAT3 (signal transducer and activator of transcription 3) signaling pathway. The STAT-3 protein is involved in the regulation of cellular processes, including the cell cycle, cell proliferation, cell apoptosis, and tumor development, while the constitutive activation of STAT-3 has been reported to be associated with a poor prognosis for various types of cancer [78,79]. Moreover, other published data showed that IL-6 is overexpressed in the resistant phenotypes of melanoma, the high levels being associated with shorter overall survival in patients receiving ICIs [66,80,81].

Regarding TNF-α expression, no significant difference was found compared to controls, nor was any other association found with any of the parameters analyzed during Nivolumab immunotherapy. The results suggest a favorable evolution of our patients, considering the role of TNF-α in stimulating tumor progression. The involvement of TNF-α in this process was supported by data obtained by a research group that showed that blocking the TNF-α receptors in an immunogenic mouse melanoma model under anti-PD-1 therapy induced the regression of melanoma in 75% of subjects [82]. Other groups showed that TNF-α induces therapy resistance by affecting the accumulation of infiltrating CD8^+^ T lymphocytes and that TNF-α transcripts were correlated with treatment failure [33,83].

In addition to the evaluation of the two soluble cytokines, the serum proteins LDH and S100, which are commonly used prognostic markers for melanoma, were also measured. After analyzing the experimental data, we found a positive correlation between the levels of LDH and both the soluble IL-6 and S100 proteins; our results are supported by the data of Hoejberg et al., which showed an almost four-fold risk of early death for patients who presented a combination of elevated levels of both serum IL-6 and LDH [84].

Recently, white-blood-cell-based inflammatory indices have become well-known and significant prognostic tumor markers due to the interconnection between the innate immune response and adaptive immunity bringing considerable benefits to melanoma patients following the homeostasis of the immune system during immunotherapy [31,56,85]. The results revealed a direct correlation between SIRI, a predictive index of response to therapy, and the soluble S100 and IL-6 proteins, biomarkers known to have a high frequency of elevated levels in melanoma patients who do not respond to therapy and are at high risk of disease progression/recurrence. These associations related to the inflammatory process and disease progression might improve the accuracy evaluation of therapy efficacy [27,86].

In terms of the limitations of this exploratory study, several aspects should be considered because of their impact on reducing the statistical power of our preliminary results and their interpretation and limiting the analyses of the predictive markers taken into account for the evaluation of the immunotherapy response. The main aspect refers to the small cohort of patients, even though the evaluation of parameters under study was performed in the dynamics of treatment and the blood samples were serially collected; therefore, a larger patient cohort is needed to enhance the statistical value of future research. Our work aimed to find biomarkers of response to Nivolumab therapy that are simple and easy to apply in daily practice and could be used to assess a response earlier than other methods can (earlier than imagistic evaluation) and not to select patients for appropriate therapy. The idea was to compare samples from responders with those from non-responders. Our data suggest that inflammatory status might predict the response to therapy, with this study still being in a hypothesis-generating phase.

The preliminary results, even if they cannot lead us towards definitive conclusions, could represent a starting point for a further high-quality prospective analysis of the effectiveness of immunotherapy in melanoma patients. Future studies, besides a larger cohort of patients, will include various treatment protocols with the combined targeting of different immune checkpoints or BRAF and MEK inhibitors, associated with clinical evidence for melanoma patients.

## 5. Conclusions

A multi-parametric evaluation during anti-PD-1 therapies in patients with advanced melanoma can show changes in normal values for some immune cells in peripheral blood, such as T-cell subsets, B cells, and NK cells, which could provide valuable information on the efficacy of the immune treatment. 

In conclusion, our findings highlight that the patients with advanced melanoma treated with Nivolumab recorded, in dynamics, an increase in the number of CD8^+^ and NK cells in 85% and 79% of cases, respectively, while CD19^+^ cells had a decreasing variation in 79% of cases, but the values varied within normal limits, indicating a positive response of the immune system following the inhibition of PD-1 function. An analysis of correlations between immune cells and inflammatory ratios/indices showed negative correlations of CD3^+^, CD4^+^, and CD19^+^ cells with NLR, and CD19^+^ with SIRI and SII, which could indicate that a low inflammatory state favors the presence and function of T and B immune cells. Tumor markers, such as LDH, established a positive correlation with IL-6 and S100 proteins, suggesting that their high levels could indicate a risk of tumor cell resistance to therapy. Blood biomarkers used to assess the immune cell response, inflammatory context, or tumor marker variation could provide valuable information about the efficacy of immune therapy, but large-scale studies of clinical evidence are needed to identify melanoma patients who respond to treatment.

## Figures and Tables

**Table 1:** Clinical data of selected patients with advanced cutaneous melanoma who underwent immunotherapy with PD-1 inhibitors.

Clinico-Pathological Characteristics		Number of Patients	Percentage (%)
Age (years)	57.5 ± 12.6 (mean ± SD)	20	
Sex	Female	13	65%
	Male	7	35%
Tumor location	Chest	13	65%
	Inferior limbs	3	15%
	Superior limbs	1	5%
	Head and neck	1	5%
	Pelvic	2	10%
Histological subtype	Superficial spreading	15	75%
	Nodular	4	20%
	Acral	1	5%
Stage	III	8	40%
	IV	12	60%

**Table 3:** Hematological data and inflammatory ratios/indices in the dynamics of PD-1 inhibitor treatment of 20 patients with advanced melanoma.

Variables	T1 (*n* = 20)	T2 (*n* = 20)	T3 (*n* = 20)	*p*-Value T1 vs. T3
Lymphocytes (×10^9^/L)	1.69 (1.40–2.15)	2.02 (1.52–2.53)	2.08 (1.51–2.50)	>0.05
Neutrophils (×10^9^/L)	3.19 (2.61–4.11)	3.85 (2.81–4.72)	3.29 (2.87–5.11)	<0.001
Monocytes (×10^9^/L)	0.37 (0.31–0.48)	0.44 (0.36–0.58)	0.52 (0.40–0.57)	<0.001
Platelets (×10^9^/L)	251.00 (216.75–293.00)	318.00 (235.75–341.75)	290.00 (252.25–351.75)	<0.001
NLR	1.74 (1.39–2.71)	2.01 (1.38–2.64)	1.55 (1.19–2.85)	<0.001
MLR	0.19 (0.17–0.33)	0.23 (0.16–0.33)	0.21 (0.15–0.34)	<0.001
PLR	145.93 (105.75–216.75)	146.50 (121.00–170.50)	135.00 (108.31–260.75)	<0.01
SII (×10^9^/L)	446.55 (330.81–662.97)	555.36 (370.61–928.60)	417.14 (315.48–966.80)	<0.001
SIRI (×10^9^/L)	0.66 (0.46–1.25)	0.72 (0.56–1.46)	0.83 (0.41–1.92)	<0.001

NLR, neutrophil-to-lymphocyte ratio; MLR, monocyte-to-lymphocyte ratio; PLR, platelet-to-lymphocyte ratio; SII, systemic immune-inflammation index; SIRI, systemic inflammatory response index; T1, T2, T3, sample collection times, at 3-month intervals, in the Nivolumab-treated melanoma group. Data are expressed as median values and quartile ranges (25–75%). The statistical significance was calculated by comparing the absolute blood count values between T1 and T3 and expressed as *p*-values.

**Table 4:** Statistical characteristics of each biomarker analyzed for the melanoma group.

Melanoma Group	SII (189–1168) *	SIRI (≤1.26) *	IL-6 (≤5.19 pg/mL) *	TNF-α (≤8.1 pg/mL) *	S100 (≤90 ng/L) *	LDH (125–220 U/L) *
Number of samples	60	60	60	60	36	60
Median	448.77	0.72	2.28	3.62	98.91	188.00
Mean	751.95	1.05	2.78	3.91	284.58	212.17
SD (±)	815.95	0.83	1.74	1.35	398.19	67.37
Minimum	105.48	0.20	1.52	0.49	29.00	142.00
Maximum	5182.01	3.43	12.40	11.77	1580.32	456.00
25th percentile (25th%)	338.16	0.45	1.89	3.42	64.54	171.75
75th percentile (75th%)	960.61	1.54	2.97	4.04	315.75	237.00

SII, systemic immune-inflammation index; SIRI, systemic inflammatory response index; IL-6, interleukin-6; TNF-α, tumor necrosis factor; LDH, lactate dehydrogenase; *, normal values.

**Table 2:** Descriptive statistical data for peripheral blood immune cells, analyzed in control vs. melanoma groups and expressed either as percentage values (**A**) or absolute counts (**B**).

(A)					
	Control Group (*n* = 20) Positive Cells (%)		Melanoma Group (*n* = 104) Positive Cells (%)		*p*- Value
Cell Types	Mean ± SD	Median (25th–75th%) ^a^	Mean ± SD	Median (25th–75th%) ^a^	
CD3^+^ T cells	72.09 ± 4.97	70.87 (68.33–75.56)	68.39 ± 13.40	70.00 (62.60–76.10)	<0.05
CD3^+^CD4^+^ T cells	45.47 ± 4.63	45.27 (41.82–48.59)	37.53 ± 9.45	37.35 (32.68–43.48)	<0.001
CD3^+^CD8^+^ T cells	30.60 ± 4.40	31.34 (28.24–33.04)	30.14 ± 11.34	28.72 (21.87–39.47)	>0.05
CD4^+^/CD8^+^ ratio	1.81 ± 0.43	1.46 (1.27–1.78)	1.48 ± 0.82	1.21 (0.85–1.95)	>0.05
CD19^+^ B cells	10.33 ± 4.01	9.26 (8.03–11.58)	13.16 ± 6.24	13.11 (7.59–17.79)	<0.01
CD16^+^CD56^+^NK cells	13.21 ± 5.53	13.20 (10.02–6.25)	16.89 ± 10.57	14.11 (9.92–21.65)	<0.05
(**B**)					
	**Control Group** **Absolute counts (×10^3^/μL)**		**Melanoma Group** **Absolute counts (×10^3^/μL)**		***p* *-*** **Value**
Cell Types	Mean ± SD	Median (25th–75th%) a	Mean ± SD	Median (25th–75th%) a	
CD3^+^ T cells	1.35 ± 0.28	1.34 (1.16–1.55)	1.36 ± 0.52	1.20 (0.94–1.78)	<0.05
CD3^+^CD4^+^ T cells	0.85 ± 0.16	0.84 (0.70–0.94)	0.72 ± 0.27	0.68 (0.51–0.89)	<0.001
CD3^+^CD8^+^ T cells	0.58 ± 0.15	0.58 (0.48–0.70)	0.63 ± 0.35	0.54 (0.35–0.77)	>0.05
CD4^+^/CD8^+^ ratio	1.81 ± 0.42	1.46 (1.27–1.77)	1.50 ± 0.85	1.22 (0.85–1.96)	>0.05
CD19^+^ B cells	0.19 ± 0.07	0.18 (0.14–0.23)	0.24 ± 0.14	0.21 (0.13–0.32)	<0.01
CD16^+^CD56^+^NK cells	0.25 ± 0.09	0.25 (0.19–0.33)	0.30 ± 0.19	0.22 (0.14–0.43)	<0.05

CD3^+^ T cells—total T lymphocytes; CD3^+^CD4^+^ T cells—helper/inducer T lymphocytes; CD3^+^CD8^+^ T cells—suppressor/cytotoxic T lymphocytes; CD4^+^/CD8^+^ ratio—helper/suppressor ratio; CD19^+^ B cells—total B lymphocytes; CD16^+^CD56^+^ NK cells—natural killer cells. ^a^—quartiles of median values (25% of the measurements are less than the lower quartile, 75% are less than the upper quartile). The statistical significance was calculated by comparing values from the melanoma group to the control group and expressed as a *p*-value.

## Data Availability

The data generated or analyzed during this study are included in the article and Supplementary Materials.

## Supplementary Materials

The following supporting information can be downloaded at https://www.mdpi.com/article/10.3390/biomedicines13061378/s1, Table S1: Flow cytometric analysis of lymphocyte-gated populations for each marker under study in peripheral blood samples from three advanced melanoma patients treated with Nivolumab, and compared to a normal sample. TableS2: Pearson’s *r* correlation and *p* statistical significance values, analyzed between immune T-cell subset, B cell, and NK cell expression levels and SIRI, SII, NLR, MLR, and PLR indices. Figure S1: Heatmap matrix of *r*-values from Pearson correlation analysis between different immune blood parameters, measured in Nivolumab-treated advanced melanoma patients.
