# Supplementary material for: Variation in Immune and Inflammatory Blood Markers in Advanced Melanoma Patients Treated with PD-1 Inhibitors: A Preliminary Exploratory Study
Source: Biomedicines. 2025 Jun 4;13(6):1378. doi: 10.3390/biomedicines13061378 (PMC12190117; doi:10.3390/biomedicines13061378)
Supplement: Supplementary file 1 [file biomedicines-13-01378-s001.zip › Table S1.pdf]

Table S1. Flow cytometric analysis of lymphocyte-gated populations for each marker under study, in peripheral blood samples from three advanced melanoma patients treated with Nivolumab, and compared to a normal sample.

Data are presented as double stained dot plots, and overlaid histograms for each immunophenotyped marker.

(A) CD3$^+$FITC/CD19$^+$PE, **(B)** CD4$^+$ FITC/CD8$^+$ PE, (**C)** CD16$^+$CD56$^+$ FITC/ CD3$^+$ PE.

Dot plot quadrants: Q1/UL (-/+) = upper-left quadrant, Q2/UR (+/+) = upper-right quadrant, Q3/LL (-/-) = lower-left quadrant, Q4/LR (+/+) = lower-right quadrant.

Overlaid histograms: Normal -------- Patient #1 -------- Patient #2 -------- Patient #3 --------

(A)

Markers	Normal	Patient #1	Patient #2	Patient #3	Normal *vs.* Patients
CD3$^+$/ D19$^+$	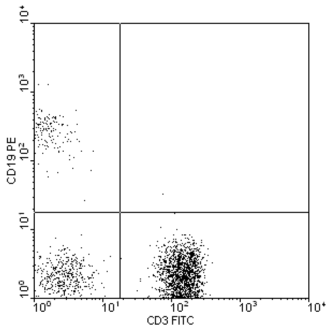	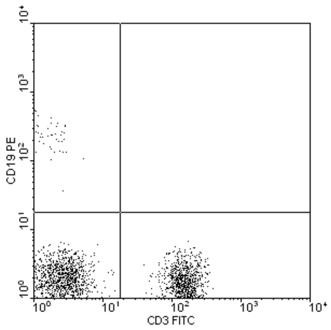	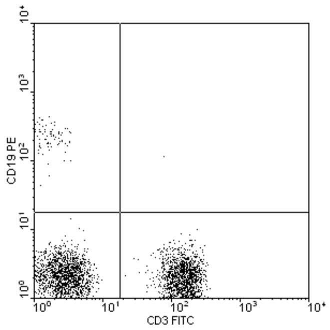	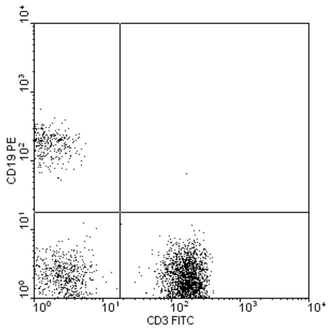	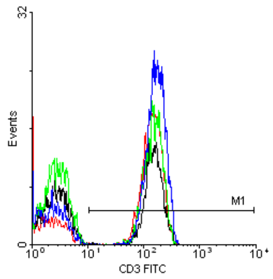
	Q1 = 11.05% Q2 = 0.03%<br>Q3 = 17.40% Q4 = 71.51%	Q1 = 4.51% Q2 = 0.00%<br>Q3 = 44.72% Q4 = 50.77%	Q1 = 6.01% Q2 = 0.03%<br>Q3 = 43.64% Q4 = 50.33%	Q1 = 14.80% Q2 = 0.03%<br>Q3 = 17.24% Q4 = 67.93%	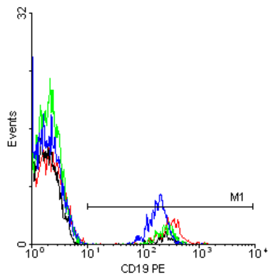

(B)

Markers	Normal	Patient #1	Patient #2	Patient #3	Normal *vs.* Patients
CD4⁺/ CD8⁺	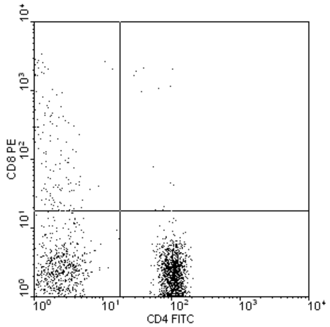	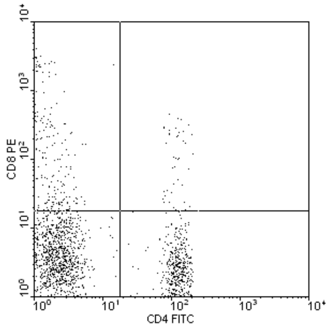	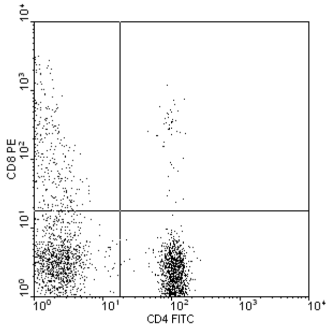	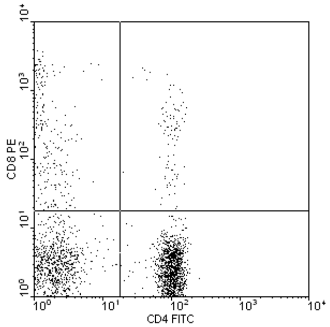	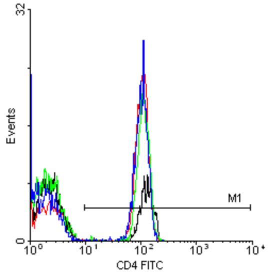
	Q1 = 23.40% Q2 = 0.45%<br>Q3 = 24.23% Q4 = 51.92%	Q1 = 33.85% Q2 = 1.66%<br>Q3 = 42.93% Q4 = 21.56%	Q1 = 27.30% Q2 = 1.28%<br>Q3 = 34.21% Q4 = 37.20%	Q1 = 35.05% Q3 = 2.45%<br>Q3 = 24.97% Q4 = 37.53%	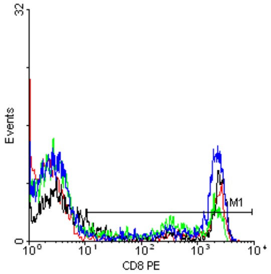

(C)

Markers	Normal	Patient #1	Patient #2	Patient #3	Normal *vs.* Patients
CD3/ CD16⁺CD56⁺	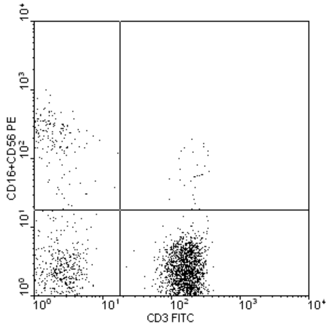	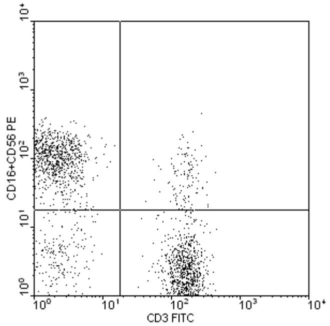	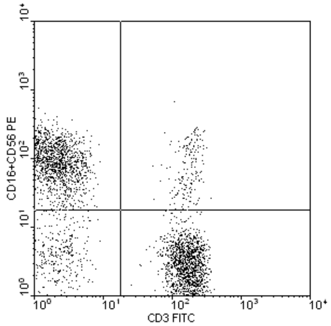	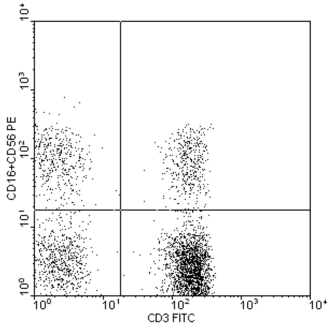	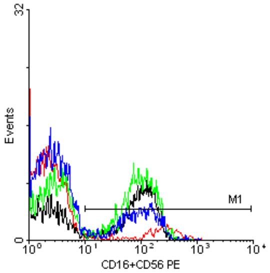
	Q1 = 11.03% Q2 = 1.01%<br>Q3 = 15.96% Q4 = 72.00%	Q1 = 45.02% Q2 = 4.68%<br>Q3 = 9.28% Q4 = 41.02%	Q1 = 41.86% Q2 = 4.62%<br>Q3 = 9.14% Q4 = 44.38%	Q1 = 13.89% Q2 = 9.98%<br>Q3 = 18.80% Q4 = 57.33%	
