# Supplementary material for: Variation in Immune and Inflammatory Blood Markers in Advanced Melanoma Patients Treated with PD-1 Inhibitors: A Preliminary Exploratory Study
Source: Biomedicines. 2025 Jun 4;13(6):1378. doi: 10.3390/biomedicines13061378 (PMC12190117; doi:10.3390/biomedicines13061378)
Supplement: Supplementary file 1 [file biomedicines-13-01378-s001.zip › Table S2 din mdpi modificat(1).pdf]

Table S2. Analyses of Pearson's correlations (*r*- values) and statistical significance (*p* – values) between percentages of immune T cells subsets, B cells, NK cells, and SIRI, SII, NLR, MLR, PLR indexes

**Variables (n= 104)**		**SIRI**	**SII**	**NLR**	**MLR**	**PLR**	**CD3$^+$**	**CD19$^+$**	**CD4$^+$**	**CD8$^+$**	**CD16$^+$CD56$^+$**	**CD4$^+$/CD8$^+$**
**SIRI**	*r*-value	—										
	p-value	—										
**SII**	*r*-value	0.868 ***	—									
	p-value	< 0.001	—									
**NLR**	*r*-value	0.834 ***	0.892 ***	—								
	p-value	< 0.001	< 0.001	—								
**MLR**	*r*-value	0.602 ***	0.410 ***	0.384 ***	—							
	p-value	< 0.001	< 0.001	< 0.001	—							
**PLR**	*r*-value	0.747 ***	0.871 ***	0.700 ***	0.479 ***	—						
	p-value	< 0.001	< 0.001	< 0.001	< 0.001	—						
**CD3$^+$**	*r*-value	- 0.230 *	- 0.168	- 0.402 ***	- 0.117	- 0.159	—					
	p-value	0.019	0.088	< 0.001	0.237	0.108	—					
**CD19$^+$**	*r*-value	- 0.316 *	- 0.315 *	- 0.334 **	0.066	- 0.190	- 0.033	—				
	p-value	0.012	0.013	0.008	0.608	0.139	0.798	—				
**CD4$^+$**	*r*-value	- 0.227 *	- 0.275 **	- 0.368 ***	0.009	- 0.177	0.475 ***	0.329 **	—			
	p-value	0.020	0.005	< 0.001	0.926	0.072	< 0.001	0.009	—			
**CD8$^+$**	*r*-value	0.011	0.121	- 0.100	- 0.088	0.017	0.585 ***	- 0.505 ***	- 0.272 **	—		
	p-value	0.909	0.222	0.312	0.372	0.862	< 0.001	< 0.001	0.005	—		
**CD16$^+$CD56$^+$**	*r*-value	0.037	0.043	0.111	- 0.191	- 0.046	-0.820 ***	- 0.498 ***	- 0.443 ***	0.003	—	
	p-value	0.777	0.738	0.391	0.136	0.721	< 0.001	< 0.001	< 0.001	0.981	—	
**CD4$^+$/CD8$^+$**	*r*-value	- 0.081	- 0.186	- 0.113	0.038	- 0.116	- 0.139	0.442 ***	0.656 ***	- 0.777 ***	- 0.220	—
	p-value	0.412	0.059	0.253	0.701	0.239	0.158	< 0.001	< 0.001	< 0.001	0.086	—

* $p < 0.05$, ** $p < 0.01$, *** $p < 0.001$
