# Supplementary figures and images for: Variation in Immune and Inflammatory Blood Markers in Advanced Melanoma Patients Treated with PD-1 Inhibitors: A Preliminary Exploratory Study
Source: Biomedicines. 2025 Jun 4;13(6):1378. doi: 10.3390/biomedicines13061378 (PMC12190117; doi:10.3390/biomedicines13061378)

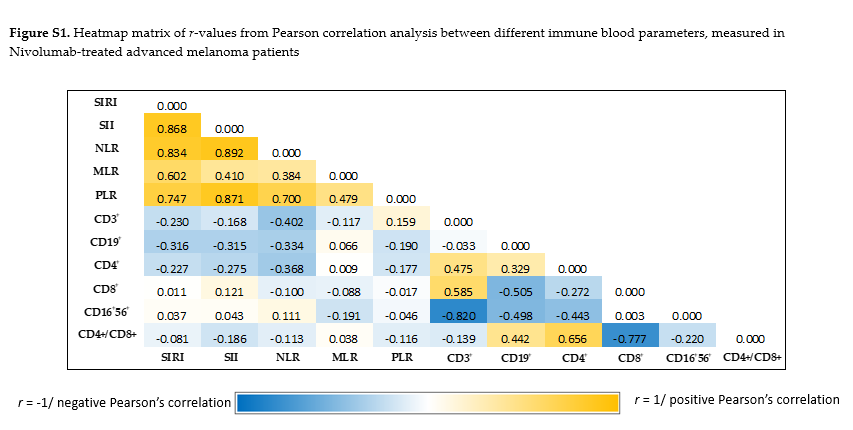

Supplement: Supplementary file 1 [file biomedicines-13-01378-s001.zip › Figure S1.tif]
